# Silkworm-derived carbon nano rods (swCNR) for detection of bismuth ions (Bi^3+^) in aquatic medium and their antiradical properties

**DOI:** 10.1016/j.heliyon.2024.e33572

**Published:** 2024-06-26

**Authors:** Neha Sharma, Anshul Sharma, Miey Park, Hae-Jeung Lee

**Affiliations:** a College of Bionanotechnology, Department of Food and Nutrition, Gachon University, Seongnam-si, Gyeonggi-do, 13120, Republic of Korea; b Institute for Aging and Clinical Nutrition Research, Gachon University, Seongnam-si, Gyeonggi-do, 13120, Republic of Korea; c Department of Health Sciences and Technology, Gachon Advanced Institute for Health Science and Technology (GAIHST), Gachon University, Incheon, 21999, Republic of Korea

**Keywords:** Green synthesis, Carbon nanorods, Silkworm, Bismuth, Metal ion, Antioxidant

## Abstract

The extensive utilization of bismuth and its derivatives in many industries, such as chemical, semiconductor, pharmaceutical, and cosmetics, leads to their accumulation in wastewater, posing a risk to both human health and the environment. Carbon nanorods (CNR) are fluorescent nanoparticles with an ability to detect various analytes as sensing probes. This study focuses on the production, structure, and chemical composition characterization of silkworm-derived CNR (swCNR) and their ability to detect bismuth ions (Bi^3+^) and inhibit radicals. The optimum wavelength for exciting the fluorescence of swCNR was 370 nm, and the resulting emission peak was observed at 436 nm. The prepared swCNR showed static fluorescence quenching mechanism-based sensing of Bi^3+^ ions with a limit of detection of 175 nM and two linear ranges from 0.5 to 5 μM (R^2^ = 0.9997) and 10–50 μM (R^2^ = 0.9995). The swCNR demonstrated high selectivity in detecting Bi^3+^ ions in the spiked river water samples, thus establishing the swCNR's role as a nano fluorescence probe designed for the selective detection of Bi^3+^ ions among other metal ions. Favorable results for the antiradical ability of swCNR were obtained against hydroxyl, 2,2 diphenyl-1 picrylhydrazyl, and 2,2′-azino-bis (3-ethylbenzothiazoline-6-sulfonic acid) radicals with scavenging percentages of 15, 32, and 90, respectively. The possible applications of swCNR in the environmental and antioxidant sectors are proposed in this study.

## Graphical abstract

**Figure:**
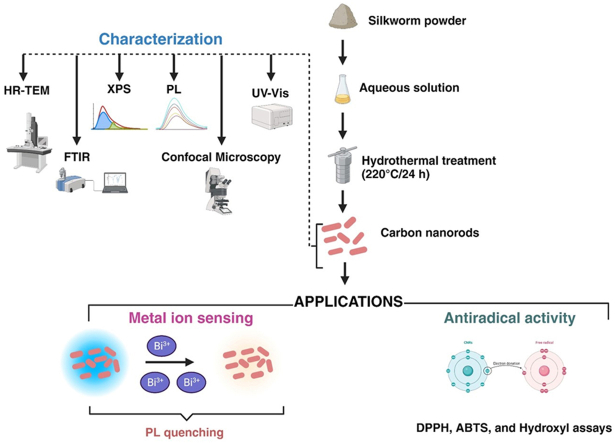

## 1 Introduction

Bismuth (Bi) is a heavy metal sometimes referred to as a "rare element" due to its limited abundance. Its application spans across various domains such as medicine, industry, cosmetics, and laboratories [1]. Bismuth and its compounds are extensively utilized in medicine as an antibacterial agent for treating *Helicobacter pylori* infections as well as related conditions such as stomach cancer, peptic ulcers, and gastritis. Additionally, they are used as an antiviral agent and for promoting burn healing [2]. These compounds are not only used in cosmetics such as lip balms, eyeliners, nail paints, and eyeshadows, but they are also utilized in the manufacturing of semiconductors and alloys, thermoelectric materials, bullets, lubricating grease, ductile steels, batteries, and several other products [1]. Although they may initially seem non-toxic, chronic use of these compounds has been proven to be detrimental to human health. This can result in elevated blood levels of Bi with symptoms of neurological damage, liver damage, kidney problems, and gastrointestinal toxicity [3]. Moreover, the utilization of household and personal items containing Bi compounds can potentially contaminate water sources, posing a serious threat to the environment [4].

Various methods have been used to detect Bi^3+^ ions, including atomic fluorescence spectrometry [5], electrochemistry using anodic striping voltammetry [3], inductively coupled plasma (ICP) atomic emission spectrometry [6], fluorescence spectrometry [7], resonance light scattering [8], ICP mass spectrometry [9], and electrothermal atomic absorption spectrometry [10]. Most of these analytical techniques have inherent limitations, such as lengthy processing times, complex instrumentation, and labour-intensive procedures. Nevertheless, the fluorescence-based method is advantageous and preferable due to its sensitivity and low detection limit for Bi^3+^ ions. Few researchers have reported the detection of Bi^3+^ ions using fluorescent probes. A study showed that the fluorescence of the tiron was quenched in the presence of Bi^3+^ions, leading to its detection [11]. Another study showed that Bi^3+^ ions selectively reduced the green fluorescence of the metal organic framework (CAU-1-(OH)_2_) probe [12]. A highly sensitive terbium chelate probe was developed to detect the Bi^3+^ ions in biological samples [13]. Qu et al. developed a fluorescent nano sensor based on carbon nanodots (CNDs) for quantifying Bi^3+^ ions in common drugs. The introduction of Bi^3+^ ions resulted in the reduced fluorescence of the yellow emitter CNDs [14]. Another study reported a pyrene-based chemosensor that exhibited increased fluorescence when exposed to Bi^3+^ ions [15]. Pyreno (4,5-d) imidazole derivatives were effectively employed as a fluorescent probe to detect Bi (III) ions in spiked real water samples [7].

Green-produced carbon dots and carbon quantum dots (CQDs) for PL-based metal ion sensing have been the subject of numerous studies [[16], [17], [18]]. However, the literature on green-produced carbon nanorods and metal sensing is scarce.

Carbon nanorods (CNRs) are 1D fluorescent nanoparticles that have been found to have potential applications in various fields such as supercapacitors [19], electromagnetic absorption [20], hydrogen storage [21], electrocatalysts for N_2_ reduction [22], dye degradation [23], and DNA detection [24]. There is only one available paper that shows the use of CNRs for detecting metal ions. The study demonstrated the capacity of 2,2′-(ethylenedioxy)-bis (ethylamine) -functionalized CNR to detect iron (III) and chromium (VI) ions [25].

Carbon nanorods can be produced using different approaches, including chemical vapor deposition [26], pyrolysis [24], electron-beam technology [27], hydrothermal [28], arc discharge process [29], solvothermal [30], and carbonization [31]. The production of water-soluble CNRs is a great challenge for the scientists, requiring the considerable utilization of chemicals in their preparation.

Our previous study showed the utilization of CNRs as potential antidiabetic agents [32]. In the present study, these water-soluble CNR are evaluated for detecting metal ions based on their intrinsic photoluminescence (PL) property. Furthermore, their ability to counteract free radicals using 2,2-azino-bis-3-ethylbenzothiazoline-6-sulfonic acid (ABTS), 2,2-diphenyl-1-picrylhydrazyl (DPPH), and hydroxyl (OH) assays has also been observed. The mechanism behind the sensitive and selective capacity of swCNR to sense Bi^3+^ ions is also discussed. To the best of our knowledge, this is the first report demonstrating Bi^3+^ ions sensing and antiradical properties by carbon nanorods.

## 2 Experimental section

### 2.1 Materials

Bismuth chloride, cobalt nitrate, zinc acetate, mercury bromide, silver nitrate, lead chloride, cadmium sulphate, copper sulphate, barium hydroxide, iron (II) chloride, iron (III) chloride, DPPH, ABTS, salicylic acid, iron chloride, hydrogen peroxide, and potassium persulfate were purchased from Sigma Aldrich. A real sample of water was taken from the Tancheon river, located in Gyeonggi-do, Republic of Korea. All reagents were utilized as obtained without further purification. Deionized (DI) water was used for all the experiments.

### 2.2 Instruments

Fluorescence measurements were performed on a Hitachi F7000 fluorescence spectrophotometer. The transmission electron microscopy (TEM) and high-resolution transmission electron microscopy (HRTEM) measurements were obtained on a Tecnai G2 F20 electron microscope. Confocal microscope Nikon (Eclipse C1si) was utilized to capture fluorescent images. The ultraviolet–visible (UV–vis) absorption spectra were obtained on a Cary 300 Bio UV–visible spectrophotometer. The Fourier transform infrared (FT-IR) spectra of the samples were analyzed by a WQF-520A FTIR spectrophotometer using KBr pellets. The antiradical activity was assessed using an Epoch Biotek plate reader at selected wavelengths. Zeta potential and dynamic light scattering spectra (DLS) were recorded using Brookhaven (Zeta PALS). Time resolved spectrometer system (PTI/Master 300) was utilized to obtain fluorescence lifetime decay (τ) measurements.

### 2.3 Synthesis and characterization of swCNR

The detailed procedure for CNR synthesis has been described previously [32]. Briefly, the silkworm powder was given hydrothermal treatment in an autoclave (Teflon-coated) for 24 h at 220 °C. The obtained solution was centrifuged at 10,000 rpm for 5 min, and its supernatant was filtered through a 0.22 μm filter and stored at room temperature (25 °C) for further applications.

### 2.4 Quantum yield (QY calculation)

The QY of swCNR was calculated by using the following equation:

(1) Q_SW_ = Q_STD_ (F_SW_ / F_STD_) (Ab_STD_/ Ab_SW_)

For measuring QY, the reference used was quinine sulphate. In equation (1), *Q* indicates QY, SW and STD represent swCNR and standard, respectively. Ab denotes absorbance and F represents the PL intensity.

### 2.5 Antiradical potential of swCNR

The radical scavenging potential of swCNR was determined by applying several antioxidant methods and calculating it using the following formula:

(2) %inhibition capacity = (Uc-Us)/Uc*100

Where U denotes the absorbance of the radicals, c stands for control, and s refers to the sample.

(swCNR or standard).

#### 2.5.1 OH scavenging activity

Fenton's reaction was used to explore the scavenging of OH free radicals. The previously reported method was used with modifications [33]. Different concentrations of swCNR were added to the mixture containing 1.8 mM ethanolic salicylic acid (375 μL) and 1.8 mM iron chloride (500 μL). Finally, after adding 25 μL of hydrogen peroxide, the mixture was incubated at 37 °C for 5 min. At 510 nm, the absorbance was measured.

#### 2.5.2 DPPH radical scavenging activity

The DPPH scavenging effectiveness of swCNR was assessed by tracking the decrease in color of methanolic DPPH (100 μM) by swCNR. swCNR was mixed with DPPH in a ratio of 1:9 and incubated in the dark for 30 min. The absorbance of the mixture was assessed at 517 nm after centrifugation at 5000 rpm for 3 min [34].

#### 2.5.3 ABTS radical scavenging activity

The ABTS scavenging ability of swCNR was determined using a previously documented method with few changes [35]. The mixture of one part of 7 mM ABTS and half part of 2.45 mM potassium persulphate was reacted for 16 h in the dark for the generation of ABTS cationic radicals. The final absorbance of the cationic ABTS was adjusted with ethanol to 0.7 ± 0.2 at 734 nm. The resulting solution reacted for 6 min with different concentrations of the sample in a ratio of 9:1, and absorbance was noted at 734 nm. To find the radicals' 50 % inhibition, the half maximal effective concentration (EC_50_) value was also computed.

### 2.6 Detection of Bi^3+^ ions

In a typical experiment, different concentrations of Bi^3+^ were mixed with 2 mL of swCNR and adjusted to a total volume of 2.5 mL with DI water. At an excitation of 370 nm, the PL spectra were recorded.

### 2.7 Interference study

The effect of various metal ions such as Bi^3+^, Ba^2+^, Cu^2+^, Cd^2^^+^, Co^2+^, Ag^+^, Hg^2+^, Zn^2+^, Pb^2+^, Fe^2+^, and Fe^3+^ on the PL of swCNR was evaluated. The concentration of all the interferents was kept at 1 mM, while for Bi^3+^ ions, a concentration of 100 μM was used for the study.

### 2.8 Real sample detection

The detection of Bi^3+^ ions was performed by adding its different concentrations (1, 10, and 50 μM) to river water. Under optimized conditions, the fluorescent assay was performed in triplicate.

## 3 Results and discussion

A one step hydrothermal method was used for the creation of swCNR from silkworm powder. No harsh chemicals or processes were utilized to produce water-soluble swCNR. The use of silkworm-derived CNRs as potential antidiabetic medicine using an animal model and the evaluation of cell viability assays in murine insulinoma and human liver hepatocellular carcinoma highlight the important application of biomass-derived CNRs in biological systems [29]. This study expands upon the application of CNRs for metal ion sensing, utilizing their inherent photoluminescent attributes and antiradical activity using various assays.

### 3.1 UV–vis and PL characterization

**Fig. 1:**
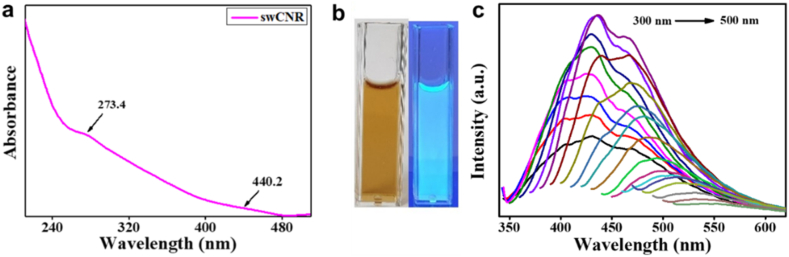
(a) Ultraviolet–Visible (UV) absorption spectra of the swCNR; (b) Photographs of swCNR under visible light and UV light; (c) Excitations dependent photoluminescence spectra of swCNR ranged from 300 to 500 nm.

The UV–Vis absorption spectrum (Fig. 1a) exhibits two peaks at 273.4 corresponding to ***π***-***π***∗ transitions and a second peak at 440.2 nm is observed due to bathochromic shift of OH atoms associated with swCNR [23]. Fig. 1b displays pictures of swCNR illuminated by natural light and UV light. As shown in Fig. 1c, the PL spectra of swCNR closely resemble the typical spectra of CNRs. The PL analysis revealed spectra at various excitation wavelengths ranging from 300 to 500 nm. The spectra may be attributed to the presence of different sized particles and surface defects existing over the surface of the swCNR.

An increase of 10 nm in the excitation wavelengths resulted in a shift in the emission spectral wavelengths. The maximum excitation and emission wavelengths of the swCNR were observed to be at 370 nm and 436 nm, respectively (Table 1). An earlier study reported the synthesis of luminescent CNRs used for the selective detection of DNA on the basis of fluorescence [24]. The observed QY of swCNR was 8.9 %. The zeta potential of swCNRs was observed to be −9.7 mV [32].

**Table 1:** Excitation dependent emission of swCNR.

S. No.	Excitation wavelength (nm)	Emission wavelength (nm)	Emission Intensity (a.u.)
1.	300	429	22482
2.	310	429	28464
3.	320	429	33670
4.	330	429	40305
5.	340	430	47841
6.	350	430	51441
7.	360	435	56382
8.	370	436	57088
9.	380	466	45670
10.	390	467	37411
11.	400	474	31076
12.	410	481	27917
13.	420	486	22111
14.	430	494	16147
15.	440	499	12458
16.	450	503	11223
17.	460	513	10870
18.	470	519	9282
19.	480	531	6476
20.	490	534	4376
21.	500	543	3494

### 3.2 Morphology and surface chemical groups study

**Fig. 2:**
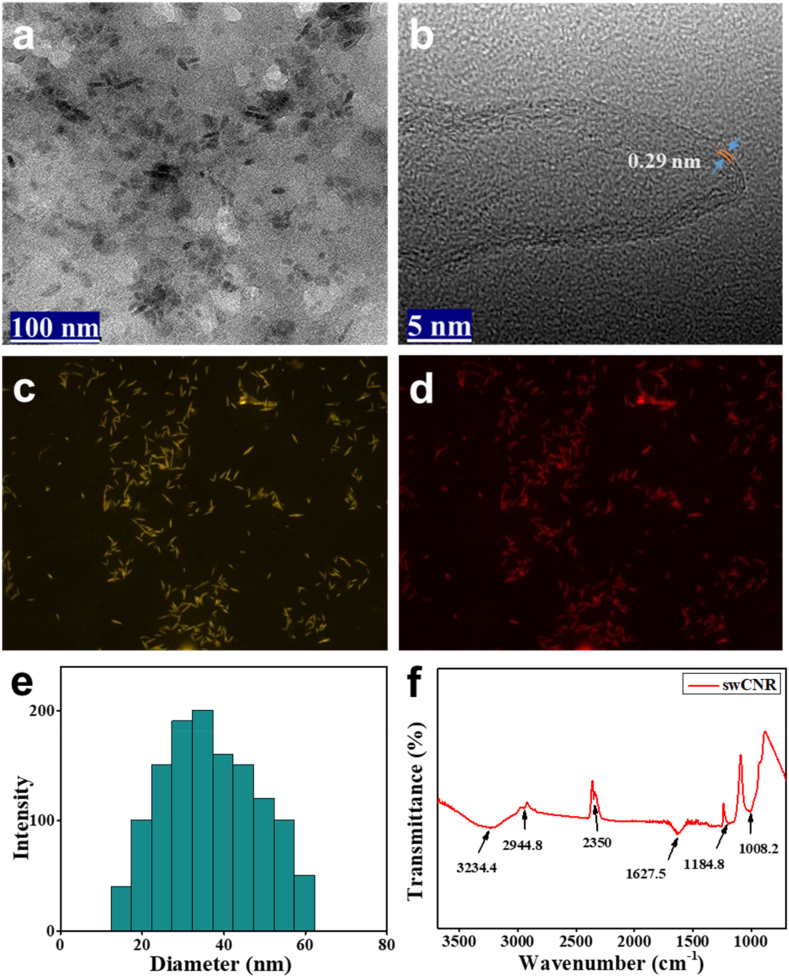
(a) Transmission electron microscope images showing rod shaped morphology of swCNR. (b) A HR-TEM image is showcasing the tip with the interlayer graphitic fringes of the swCNR (*d* = 0.29 nm). Confocal images of swCNR showing (c) yellow and (d) red color at different excitation wavelengths (λ_ex_: 405 nm or λ_ex_: 457 nm); (e) Dynamic light scattering (DLS) analysis for particle size and distribution of swCNR. (f) FTIR of swCNR. swCNR: silkworm-derived carbon nanorod; HR-TEM: high resolution transmission electron image in microscope; FTIR: Fourier-transform infrared spectroscopy.

The rod morphology of swCNR was determined through the application of TEM and confocal microscopy. The swCNR has dimensions in the nanoscale range for both its width and length. Fig. 2a shows the rod shaped morphology of the swCNR. The high resolution TEM image in Fig. 2b exhibits the sharp tip of the swCNR, revealing crystal lattice fringes with a spacing of 0.29 nm. The swCNR had an average length of 40 nm and an average diameter of 13 nm. Fig. 2c and d, display the confocal images of swCNR in yellow and red color, respectively. These images were obtained by exciting the sample at wavelengths of 405 or 457 nm, which further confirms the rod-like structure of swCNR.

DLS spectra, as shown in Fig. 2e, calculated the average particle size of the prepared swCNR to be between 15 and 60 nm in the aqueous solution. The results obtained via DLS do not distinctly attribute to one dimension (length or diameter) of the carbon nanorods but instead reflect an integrated measurement. The average size found in DLS spectra is bigger than the average size found in TEM results. This is because DLS measures the overall hydrodynamic dimensions of adsorbed molecules and ions [36].

The chemical composition and functional groups of the swCNR were obtained using FTIR (Fig. 2f). The absorption bands obtained at 1008.2, 1184.8, 1627.5, 2350, and 2944.8 cm^−1^ were ascribed to C=C bending vibrations, C–H, C=O, C≡C & C≡N, and C–H stretching, respectively. The band observed at 3234.4 cm^−1^ is associated with the O–H stretching vibration of the hydroxyl group [37].

**Fig. 3:**
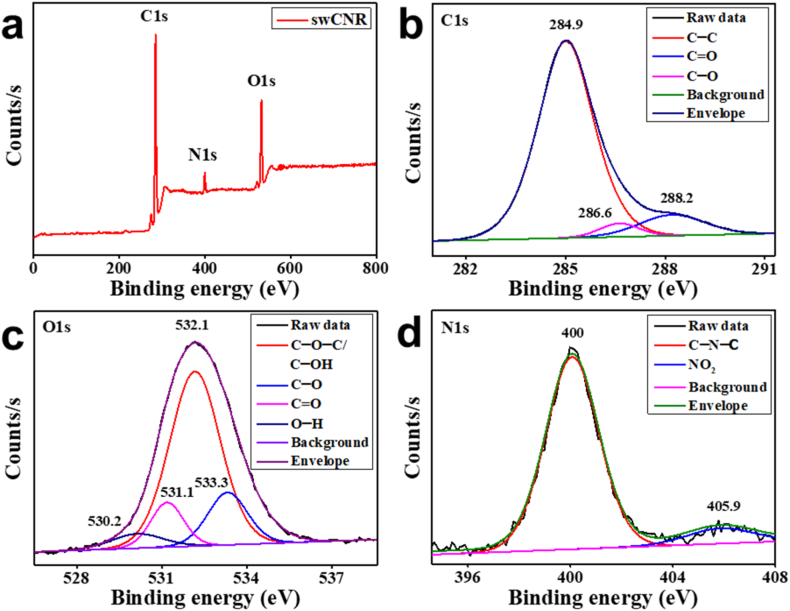
XPS spectra of the swCNR. (a) XPS Survey spectra of swCNR. Deconvoluted spectra of (b) C1s, (c) O1s, and (d) N1s. swCNR: silkworm-derived carbon nanorod; XPS: X-ray photoelectron spectroscopy.

XPS was employed to gain insight into the chemical state and surface composition of the swCNR. The survey spectra of swCNR revealed the presence of carbon (80.51 %), oxygen (13.25 %), and nitrogen (5.03 %) on its surface (Fig. 3a). The deconvolution of C1s resulted in 3 peaks at 284.9, 286.6, and 288.2 eV ascribed to C–C, C–O [38], and C=O [39] bonds, respectively (Fig. 3b). The O1s spectra is further expanded into 530.2, 531.1, 532.1, and 533.3 eV peaks which are related to the O–H, C=O, C–*O*–C/C–OH [40], and C–O bands, respectively, as shown in Fig. 3c. The two N1s peaks are assigned to the C–N–C bond at 400 eV [41] and the NO_2_ bond at 405.9 eV (Fig. 3d) [42].

### 3.3 Antioxidant potential of swCNR

The antioxidant properties of swCNR were investigated utilizing different radical scavenging assays, specifically targeting OH, DPPH, and ABTS radicals. The antioxidant capacity was assessed by using three different methods. This is because relying on a single approach is insufficient to accurately evaluate the antioxidant power of any material [43]. The results were compared with standard l-ascorbic acid.

Many studies have documented the antioxidant properties of green-synthesized nanocarbons [[44], [45], [46], [47]]. Nanocarbons such as carbon dots belong to a group of nonenzymatic radical scavengers. The antioxidant activity of nanocarbons is dependent on different pathways. Some examples of these processes are the formation of adducts with radical species in areas with a lot of sp^2^ domains, the movement of hydrogen from functional groups on the surface, and the transfer of electrons. The antiradical action of nanocarbons is contingent upon the specific type of radical as well as the composition and structure of the nanocarbons [48]. Furthermore, the composition of the media affects any material's antioxidant activity. More recently, carbon dots prepared from tea wastes showed antioxidant properties both in aqueous and oil media [44]. Moreover, nanocarbons as free radical scavengers and reactive species generator have also been documented [48,49]. An essential measure of a material's antioxidant efficacy is its redox potential (a thermodynamic quantity), which establishes its structural and activity relationship [50]. Cyclic voltammetry evaluates antioxidant activity by using redox potential. It has numerous advantages over traditional chemical assays, including its simplicity and speed and can be utilized for preliminary screening of antioxidants based on reducing power [51]. Similar technique was used by researchers for evaluating the radical scavenging capacity of nanocarbons [52].

Therefore, the antioxidant activity of nanocarbons is not only affected by their structural composition and functional attributes but on other parameters also.

**Fig. 4:**
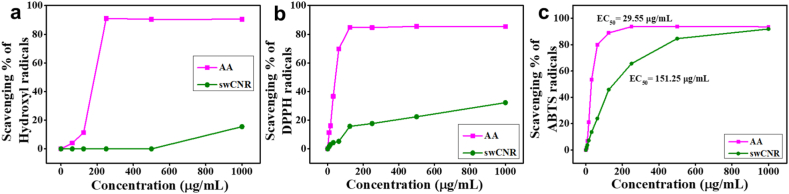
Radical scavenging capacity of swCNR and AA against (a) Hydroxyl radical, (b) DPPH radical and (c) ABTS radicals. AA: l-Ascorbic acid; ABTS: 2,2-azino-bis-3-ethylbenzothiazoline-6-sulfonic acid; swCNR: silkworm-derived carbon nanorod; DPPH: 2,2-diphenyl-1-picrylhydrazyl.

The Fenton reaction is a widely accepted method for evaluating the antioxidant potential of materials by scavenging OH radicals. Hydroxyl radicals undergo a reaction with salicylic acid, resulting in the formation of 2,3-dihydroxybenzoic acid and show absorbance at 510 nm [53]. In this study, at a concentration of 1 mg/mL, swCNR demonstrated a 15 % inhibition of OH radicals, whereas the standard scavenged 90 %, as shown in Fig. 4a. Comparable results have been documented previously [33]. At concentrations below 1 mg/mL, swCNR showed almost negligible OH inhibition ability. Mechanistically, the functional groups present on the surface of swCNR may donate hydrogen to the neutralization of OH radicals [47].

The DPPH radical scavenging capacity of swCNR is illustrated in Fig. 4b. Visually, upon the addition of antioxidants, the change of the violet color of DPPH to colorless confirms its scavenging [34]. The potential mechanism for radical scavenging involves the transfer of hydrogen from the surface groups of swCNR to DPPH. The hydroxyls, carboxyls, and amino from swCNR surface groups facilitate the transfer of hydrogen and the reduction of DPPH radical to DPPH-H. Furthermore, the rearrangement of chemical bonds and resonance within the aromatic domains can result in the delocalization of unpaired electrons across the surface of swCNR [34,48].

The presence of swCNR at a concentration of 1 mg/mL resulted in the scavenging of 32 % of DPPH radicals, whereas the standard achieved 85 % scavenging at the same concentration. Comparable potential for neutralizing DPPH radicals was obtained by Naik et al. In the study, carbon dots prepared from *Andrographis paniculata* were able to neutralize 35 % of DPPH radicals at 960 μg/mL [54]. The DPPH scavenging activity of 23 % by *Ananas comosus* derived CQDs was reported by another research group at a concentration of 8 mg/mL [46].

The ABTS method is generally employed to study the antioxidant behavior of compounds that disrupt chains and donate hydrogen atoms [55]. The mechanism underlying ABTS radical neutralization and discoloration is the transfer of electrons or hydrogen from swCNR to positively charged ABTS radicals, which transforms them into stable neutral molecules [56]. The swCNR demonstrated excellent scavenging capacity against ABTS radicals by effectively decolorizing blue green ABTS solution. Both swCNR and standard exhibited an ABTS radical inhibition ability of over 90 % at the highest concentration of 1 mg/mL (Fig. 4c). The study shows that swCNR showed a dose-dependent increase in antioxidant capacity. The EC_50_ value of swCNR was calculated to be 151.25 μg/mL. Carbon dots synthesized from turmeric with sulfur doping showed an antiradical activity of 70 % against ABTS radicals [45]. Among all methods tested, stronger antiradical activity was observed for ABTS assays, which could be due to the better dispersion and hydrophilic nature of the swCNR [45]. Consistent with our results, higher ABTS radical scavenging activity compared to DPPH radicals has been reported in earlier findings [45,57].

Antioxidants neutralize free radicals by providing an electron or hydrogen atom. It has already been reported that the presence of hydroxyl, carboxyl, and amine groups significantly contributes to the elimination of radicals [58]. Hence, swCNR comprises multiple functional groups that are accountable for its anti-radical properties.

### 3.4 Bismuth (Bi^3+^) ions detection

**Fig. 5:**
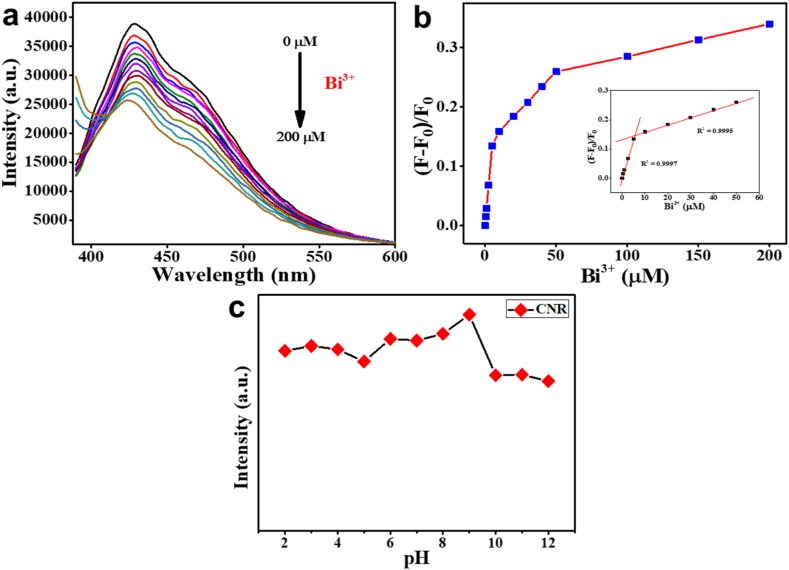
(a) Photoluminescence spectra of swCNR in the presence of Bi^3+^ ions at different concentrations (0, 0.5, 1, 2.5, 5, 10, 20, 30, 40, 50, 100, 150, and 200 μM), (b) The Stern–Volmer plot of the quenching of the fluorescent intensity of swCNR by bismuth (Bi^3+^) ions, (c) Effect of pH on the detection of Bi^3+^ ions by swCNR.

In order to investigate the sensing capability of swCNR, the PL intensity of the CNRs was measured following the addition of different concentrations of Bi^3+^ ions. Fig. 5a illustrates that the introduction of Bi^3+^ ions at concentrations ranging from 0 to 200 μM leads to a decrease in the PL intensity of swCNR. From the Stern–Volmer plot of F–F_0_/F_0_ (Fig. 5b) with various concentrations of Bi^3+^ ions, two good linear ranges were obtained from 0.5 to 5 μM and 10–50 μM. The correlation coefficients of these ranges were determined to be 0.9997 and 0.9995, respectively. The limit of detection (LOD) was calculated to be 175 nM using the universally accepted formula (3σ/s), which is comparable to the previously reported CQDs based fluorescent sensor [59].

### 3.5 Optimization of experimental conditions

To explore the effect of pH variations on the PL intensity of swCNR for the detection of Bi^3+^ ions at 100 μM, a fluorescent assay was performed. Fig. 5c shows that there was minimal variation in the PL intensity of swCNR when mixed with Bi^3+^ across a pH range of 2–8. Observations revealed that CNR had the highest PL intensity after reacting with Bi^3+^ ions at pH of 9. However, the intensity dropped when the pH was raised to alkaline levels between 10 and 12. This demonstrates that swCNR has the ability to detect Bi^3+^ ions across a wide pH range.

### 3.6 Mechanism

**Fig. 6:**
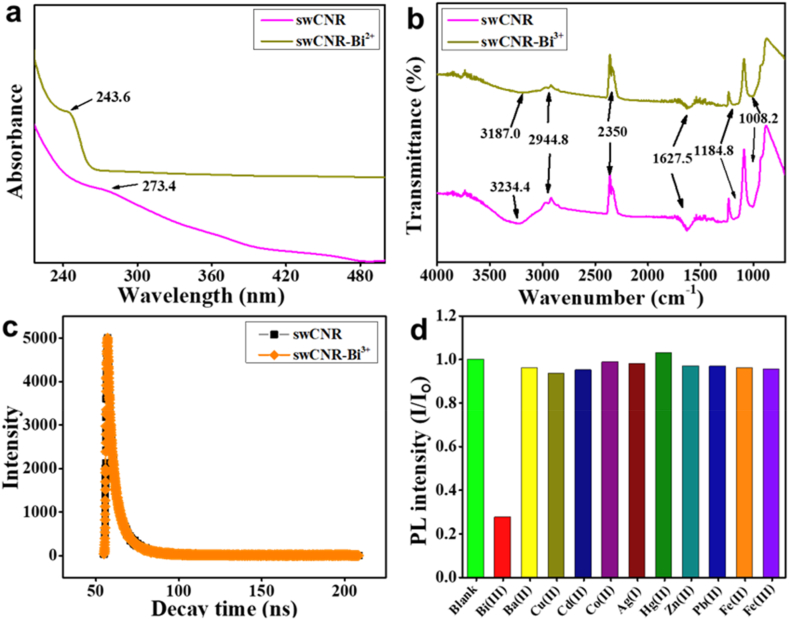
Mechanism of Bi^3+^ions detection by swCNR (a) Effect on the UV absorbance and (b) FTIR spectra of swCNR in the presence of Bi^3+^ ions. (c) Fluorescence lifetime (τ) decay curve of swCNR without and with Bi^3+^ ions. (d) Selectivity investigation of swCNR in presence of blank and various metal ions (Bi^3+^, Ba^2+^, Cu^2+^, Cd^2^^+^, Co^2+^, Ag^+^, Hg^2+^, Zn^2+^, Pb^2+^, Fe^2+^, and Fe^3+^). swCNR: silkworm-derived carbon nanorod; FTIR: Fourier-transform infrared spectroscopy.

In order to assess the mechanism by which swCNR detects Bi^3+^ ions, the change in the UV spectra of swCNR was recorded following the addition of Bi^3+^ ions. As depicted in Fig. 6a, the distinctive absorption peak of swCNR at 273.4 nm disappeared and shifted to 243.6 nm. This suggests that the detection of Bi^3+^ ions may be due to static quenching, which is characterized by the formation of a complex between the two substances [60]. Additionally, as shown in Fig. 6b, FTIR analysis was conducted on swCNR both with and without Bi^3+^ ions. Following the addition of Bi^3+^ ions, a reduction in the intensity of peaks at 1008.2, 1184.8, 1627.5, 2350, and 2944.8 cm^−1^ was observed. The broad O–H stretch at 3234.4 cm^−1^ displayed a little shift to 3187.0 cm^−1^ and a drop in intensity upon the addition of Bi^3+^ ions.

The lifetime decay time (τ) of swCNRs without and with Bi^3+^ ions was also evaluated using the TCSPC method to get more insight into the quenching mechanism. The shift in the lifetime of swCNR was recorded to be 5.98 ns from 6.0 ns after the addition of Bi^3+^ ions (Fig. 6c), The addition of Bi^3+^ ions did not make a significant change in the lifetime of swCNR, which suggests the occurrence of a static quenching mechanism [16]. Further, using the given Stern-Volmer equation, a quenching mechanism was explored.

(3) F_0_/F = 1+K_sv_ [Q]

Where F and F_0_ represent the intensity of photoluminescence of swCNR after and before the addition of Bi^3+^ ions, respectively. K_sv_ refers to the quenching constant of the Stern-Volmer plot. Q denotes the concentration of Bi^3+^ ions. K_q_ is the biomolecular quenching constant and can be calculated using the following equation:

(4) K_q_ = K_sv_/τ_0_

Wherein τ_0_ is the lifetime decay time of swCNRs without Bi^3+^ ions. Using τ_0_ value, the K_q_ was calculated to be 4.3 × 10^13^ L mol^−1^s^−1^. The standard value for diffusion-controlled reactions is about 1 × 10^10^ L mol^−1^s^−1^ [17]. Hence, the increased value of the k_q_ measured here suggests that a static mechanism is contributing to Bi^3+^ ion sensing by swCNR.

Overall, it was deduced that the binding interaction between Bi^3+^ ions and swCNR was responsible for the detection of Bi^3+^ ions. Prior research by Gao et al. has established that Bi^3+^ ions have the ability to form bonds with the hydroxyl and carbonyl groups of the quencher. Additionally, these ions can disrupt π bonds of CQDs, allowing their detection by CQDs [59].

### 3.7 Interference study

The selectivity of swCNR in detecting Bi^3+^ ions in comparison to other metal ions, including Ba^2+^, Cu^2+^, Cd^2^^+^, Co^2+^, Ag^+^, Hg^2+^, Zn^2+^, Pb^2+^, Fe^2+^, and Fe^3+^ was examined. Fig. 6d demonstrates that the PL of swCNR was not significantly affected by other metal ions. Therefore, swCNR demonstrated a high degree of selectivity and sensing ability for Bi^3+^.

### 3.8 Detection of Bi^3+^ in real sample

To evaluate the practical applicability of swCNR, Bi^3+^ ions was analyzed in spiked real samples of water from the river. Different concentrations of Bi^3+^ ions were quantified in the spiked river water, as shown in Table 2. The recoveries varied from 99.8 to 102.66 %, indicating the excellent ability of swCNR to be utilized as a sensor for Bi^3+^ ions detection in actual samples.

**Table 2:** Results of the recovery of Bi^3+^ ions in river water sample.

S. No.	Added Bi^3+^ (μM)	Found Bi^3+^ (μM)	Recovery (%)
1	1	0.998	99.8
2	10	10	100
3	50	51.33	102.66

### 3.9 Conclusions

Hydrothermally, swCNR were synthesized using powder of silkworm. The obtained swCNR contained an uneven outer surface with average 60 nm length and a width of 13 nm. The current study revealed that the produced nanoparticles could scavenge radicals, indicating the possibility of using swCNR as an antioxidant. Compared to the other two radicals, swCNR was found to be more efficient at eliminating ABTS radicals. swCNR efficiently removed almost 90 % of ABTS, which was comparable to the standard ascorbic acid. The presence of hydroxyl and amine groups on the surface of swCNR bestowed them with the ability to quench Bi^3+^ ions selectively in comparison to different metal ions. This work presented the applicability of swCNR as a radical scavenger and probe for Bi^3+^ ions sensing.

## Data availability

All data generated or analyzed during this study are included in this published article.

## Ethical approval and consent to participate

Not applicable.

## Consent for publication

The content of the manuscript has been approved by all the authors.

## CRediT authorship contribution statement

**Neha Sharma:** Writing – original draft, Visualization, Methodology, Investigation, Formal analysis. **Anshul Sharma:** Writing – review & editing, Formal analysis, Conceptualization. **Miey Park:** Resources. **Hae-Jeung Lee:** Supervision, Resources, Funding acquisition, Conceptualization.

## Declaration of competing interest

The authors declare that they have no known competing financial interests or personal relationships that could have appeared to influence the work reported in this paper.
